# Innate Immune Sensing of Flaviviruses

**DOI:** 10.1371/journal.ppat.1003541

**Published:** 2013-09-12

**Authors:** Mehul S. Suthar, Sebastian Aguirre, Ana Fernandez-Sesma

**Affiliations:** 1 Department of Pediatrics and Children's Healthcare of Atlanta and Emory Vaccine Center, Emory University School of Medicine, Atlanta, Georgia, United States of America; 2 Department of Microbiology and the Emerging Pathogens Institute, Icahn School of Medicine at Mount Sinai, New York, New York, United States of America; University of Florida, United States of America

## Overview

**Figure 1:**
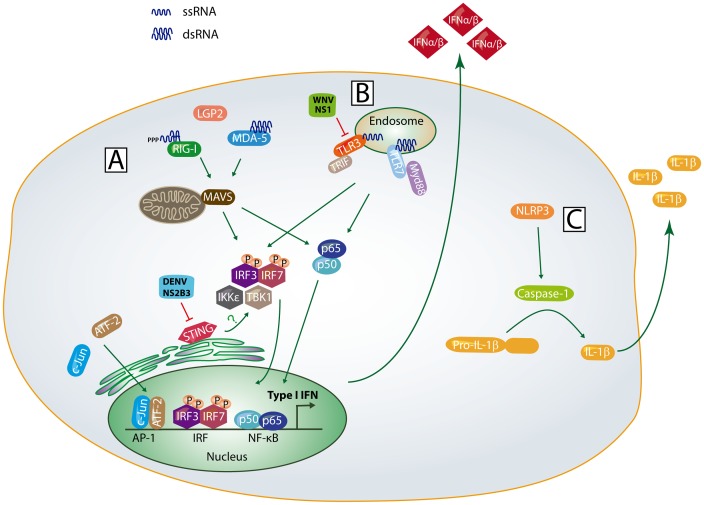
Innate immune sensing pathways. (A) Following non-self RNA binding, RIG-I and MDA5 undergo a conformational change, multimerize, and interact with MAVS through CARD-CARD interactions. This activates the adaptor STING and latent transcription factors IRF-3, IRF-7, and NF-κB to induce expression of IFN-β and IRF-3 target genes; (B) Within the endosomal compartment, TLR3 and TLR7 bind to non-self RNA, interact with their adaptor proteins, and activate IRF-3, IRF-7, and NF-κB dependent gene expression; (C) Upon detection of viral dsRNA or cellular stress signals, the NLPR3 oligomerizes, interacts with ASC, leading to activation of caspase-1 and IL-1β processing. The signaling events and host factors targeted by WNV and DENV proteins are shown.

Mosquito-borne flaviviruses cause annual epidemics of encephalitis and viscerotropic disease worldwide. West Nile virus (WNV), a member of the Japanese Encephalitis virus (JEV) antigenic complex, is a major cause of viral encephalitis throughout the world [1]. In the United States, WNV has been estimated to cause more than 3 million infections, resulting in over 780,000 illnesses, 30,000 confirmed cases, and 1,100 deaths between 1999–2013 [2]. In contrast, dengue virus (DENV) is a major cause of virus-induced viscerotropic disease, with an estimated 50 to 100 million people infected each year and a total of 2.5 billion people worldwide are at risk of infection. There are currently no approved antiviral therapies or vaccines to combat or prevent WNV or DENV infection. WNV and DENV are closely related flaviviruses that are enveloped and possess a single strand positive-sense RNA genome of approximately 11 kb in length. The genome is comprised of three structural proteins (C, prM/M, and E), which mediate virus attachment, entry, and encapsidation and seven non-structural proteins (NS1, NS2A, NS2B, NS3, NS4A, NS4B, and NS5), which form the replication complex to synthesize viral RNA (as reviewed in [3]). The NS proteins, including the viral RNA-dependent RNA polymerase NS5, form a replication complex that synthesizes negative-sense RNA intermediates, which subsequently serve as the template for synthesis of positive-sense RNA. The newly synthesized viral RNA is encapsidated, transported through the host secretory pathway, and released from the infected cell by exocytosis. The entire viral life cycle takes place within the cytoplasm. To combat infection, the host encodes pattern recognition receptors (PRR), residing within the cytoplasm and endosomal vesicles, which recognize pathogen-associated molecular patterns (PAMPs) and trigger inflammation and antiviral immune responses (Figure 1). Defining how viruses are sensed and the antiviral programs that are initiated to restrict virus replication have been areas of intense investigation over the past several years. In this article, we discuss contemporary research findings on how flaviviruses are “sensed” by the host pattern recognition receptors and highlight a few outstanding questions on the mechanisms underlying host immune sensing and viral countermeasures.

## Do Flaviviruses Activate the RIG-I Like Receptors?

The retinoic-acid inducible gene-I (RIG-I)-like receptors (RLRs) are cytosolic innate immune PRRs that recognize non-self RNA signatures, interact with the mitochondrial antiviral signaling protein (MAVS), and induce type I interferon (IFN), proinflammatory cytokines, and expression of IFN-stimulated genes (ISGs) (as reviewed in [4]). RIG-I and myeloma differentiation factor 5 (MDA5) are critical for detecting and responding to WNV, DENV, and other mosquito-borne flaviviruses [5], [6]. WNV infection of *RIG-I^−/−^* or *MDA5^−/−^* mouse embryonic fibroblasts revealed that RIG-I is activated early during infection whereas MDA5 is required for enhancing and sustaining type I IFN and ISG expression in response to infection [6]. Furthermore, *in vivo* studies have demonstrated that RLR signaling is required for protection as well as controlling peripheral organ and central nervous system (CNS) viral burden, limiting virus-mediated CNS pathology, and programming protective immunity to WNV infection [7], [8]. Similarly, RIG-I, MDA5 and MAVS, and the adaptor protein stimulator of the IFN gene (STING) are required for mediating antiviral host signaling in response to DENV infection [9], [10]. Although these receptors have been implicated in detecting and responding to flavivirus infection, it is not yet clear as to what accounts for the temporal activation of RIG-I and MDA5. Moreover, it will be interesting to determine whether the individual RLRs are functionally redundant or direct distinct antiviral programs during flavivirus infection.

## What Is the Nature of the RIG-I and MDA5 PAMP?

Biochemical and molecular analysis of RIG-I and MDA5 has revealed important RNA signatures that are critical for engagement and activation of signaling. RIG-I engages short double-strand RNA (dsRNA) possessing a 5′ triphosphate and uridine- or adenosine-rich motifs (as reviewed in [4]). MDA5, on the other hand, does not require a 5′ triphosphate but preferentially engages long dsRNA. Flavivirus contain genomic RNA contains a 5′ cap structure,, which methylated, which likely prevents RIG-I recognition of the incoming viral RNA following infection. Indeed, Fredericksen and colleagues have shown that the incoming genomic RNA is not capable of activating RLR signaling but viral RNA synthesis is required for triggering IRF-3 activation, suggesting that RIG-I and MDA5 likely engage replication intermediates generated during viral RNA synthesis [11]. In support of this hypothesis, Shipley and colleagues have recently determined that *in vitro* transcribed RNAs corresponding to distinct regions within the positive- and negative-sense WNV RNA can trigger RIG-I activation [12], although the precise immunostimulatory nucleic acid sequence or motif has not yet been determined. During viral RNA synthesis, the positive-sense RNA strand is believed to cyclize through long distance interactions between conserved sequences within the 5′ and 3′ untranslated regions [13]. It is possible that RIG-I or MDA5 may directly interact with this viral RNA structure. Alternatively, this could be a source of dsRNAs containing a 5′ triphosphate, possibly from nascent negative-sense RNAs, or a source of long dsRNAs from newly synthesized full-length positive-sense and negative-sense viral RNAs. It is also possible that genomic RNA degradation products [14] or RNaseL cleavage products [15] promote RIG-I activation during flavivirus infection. Nonetheless, further investigation is warranted to determine the exact flavivirus PAMP RNA substrate and nucleic acid sequence that is engaged by either RIG-I and MDA5.

## How Do Flaviviruses Counteract RLR Signaling?

WNV, and likely DENV, passively evade RLR activation at early times during infection [11]; however, the underlying mechanisms are not well understood. During flavivirus infection, RNA replication complexes are found within vesicle pockets, which contain nonstructural viral proteins, *de novo* synthesized RNA, and dsRNA intermediates [16]–[18]. It is possible that early during infection, these compartments sequester viral RNA replication intermediates to avoid activating RIG-I or MDA5 signaling. At later times during infection, the abundance of newly synthesized viral RNA, combined with a loss of compartment integrity, likely leads to RLR activation and innate immune signaling [11]. Unlike WNV, DENV actively inhibits type I IFN production in DCs and monocytes [19]. The viral NS2B3 protease, which normally processes the viral polyprotein, directly cleaves and degrades human STING, thus rendering RLR signaling inactive. Murine STING cannot be degraded by the DENV NS2B3 protease and could be a strong restriction factor for DENV replication in mice [10]. It is not yet clear whether STING cleavage is unique to DENV infection or a common mechanism employed by other flaviviruses. These studies also raise the intriguing possibility that other host proteins could also be targets of virus-mediated cleavage.

## What Role Do the TLRs Play in Immunity to Flavivirus Infection?

The toll-like receptors (TLRs) reside within endosomal vesicles, and upon binding PAMP RNA (TLR3- dsRNA, TLR7-ssRNA), induce type I IFN and proinflammatory cytokine expression [20]. TLR3, TLR7, and the Myd88 adaptor protein are required for protection against WNV infection (as reviewed in [3]). Although the exact viral PAMP has not yet been identified, *in vivo* studies with WNV have shown that TLR3 and TLR7 are important for promoting CNS immunity and controlling virus replication. In response to DENV infection, experimental evidence also suggests that TLR3 is important in restricting virus replication [21]. WNV NS1, which is secreted from infected cells, is believed to antagonize TLR3 signaling [22]. This finding, however, is controversial as a follow-up study by Bartoni and colleagues failed to observe inhibition of TLR3 signaling by NS1 protein from WNV, DENV, or Yellow Fever virus [23]. Overall, the flavivirus-host interactions with the TLRs are poorly understood and further investigation is needed to better understand the cell autonomous role of TLR signaling as well as determine whether flaviviruses directly antagonize TLR signaling.

## Is the Inflammasome Important?

The Nod-like receptors (NLRs) detect “danger signals” within the cellular environment, leading to formation of the inflammasome signaling complex and secretion of pro-inflammatory cytokines of the IL-1β family, including IL-1β, IL-18, and IL33 (as reviewed in [24]). WNV and DENV infection of humans is associated with elevated levels of systemic IL-1β, suggesting that viral infection activates inflammasome signaling [25]–[27]. Indeed, the NLRP3 inflammasome is activated following WNV and DENV infection [25], [28]. However, the exact danger signal that activates “signal 1,” which is responsible for triggering pro-IL-1β expression, or “signal 2,” which promotes IL-1β maturation, is not well understood. Further studies are needed to determine what role, if any, viral dsRNA or virus-induced cell stress signals trigger activation of the NLRP3 inflammasome during WNV and DENV infection.

During WNV infection, IL-1β is believed to mediate protection through several mechanisms, including regulating CD8^+^ T cell effector functions [25], regulating DC immune responses within the brain [29], controlling neuronal cell death [30], and triggering antiviral effector gene expression in neurons [25]. With relation to DENV, macrophages are believed to be the primary source of IL-1β production; however, much remains to be understood about IL-1 signaling in the context of DENV infection [28].

## Summary

Emerging mosquito-borne flavivirus infections of humans continue to be a significant problem worldwide. It is becoming increasingly evident that development of effective vaccines, which provide life-long sterilizing immunity to viral infection, requires a proper understanding of how the host innate immune sensing pathways detect viral infection and program protective immunity. Continued studies to understand the flavivirus-host interactions with PRR pathways will provide valuable insights to overcome current challenges that have hindered effective vaccine development and prophylactic treatment strategies to prevent or combat WNV and DENV infection.
